# MMADHC premature termination codons in the pathogenesis of cobalamin D disorder: Potential of translational readthrough reconstitution

**DOI:** 10.1016/j.ymgmr.2021.100710

**Published:** 2021-01-27

**Authors:** Leire Torices, Javier de las Heras, Juan Carlos Arango-Lasprilla, Jesús M. Cortés, Caroline E. Nunes-Xavier, Rafael Pulido

**Affiliations:** a Biocruces Bizkaia Health Research Institute, Barakaldo, Spain; b Division of Pediatric Metabolism (CIBER-ER), Cruces University Hospital, Barakaldo, Spain; c Department of Pediatrics, University of the Basque Country (UPV/EHU), Leioa, Spain; d Department of Cell Biology and Histology, University of the Basque Country (UPV/EHU), Leioa, Spain; e Ikerbasque, The Basque Foundation for Science, 48013 Bilbao, Spain; f Institute of Cancer Research, Oslo University Hospital Radiumhospitalet, N-0424 Oslo, Norway

**Keywords:** Methylmalonic aciduria, Homocystinuria, cblD, MMADHC, Translational readthrough, Premature termination codon, Stop codon

## Abstract

Mutations in the *MMADHC* gene cause cobalamin D disorder (cblD), an autosomal recessive inborn disease with defects in intracellular cobalamin (cbl, vitamin B12) metabolism. CblD patients present methylmalonic aciduria (MMA), homocystinuria (HC), or combined MMA/HC, and usually suffer developmental delay and cognitive deficits. The most frequent *MMADHC* genetic alterations associated with disease generate MMADHC truncated proteins, in many cases due to mutations that create premature termination codons (PTC). In this study, we have performed a comprehensive and global characterization of MMADHC protein variants generated by all annotated *MMADHC* PTC mutations in cblD patients, and analyzed the potential of inducible translational PTC readthrough to reconstitute MMADHC biosynthesis. MMADHC protein truncation caused by disease-associated PTC differentially affected the alternative usage of translation initiation sites, protein abundance, and subcellular localization of MMADHC. Aminoglycoside compounds induced translational PTC readthrough of MMADHC truncated variants, allowing the biosynthesis of full-length MMADHC in a PTC-specific manner. Our results suggest that translational PTC readthrough-based interventions could complement current therapies for cblD patients carrying specific MMADHC PTC mutations.

## Graphical abstract

**Figure:**
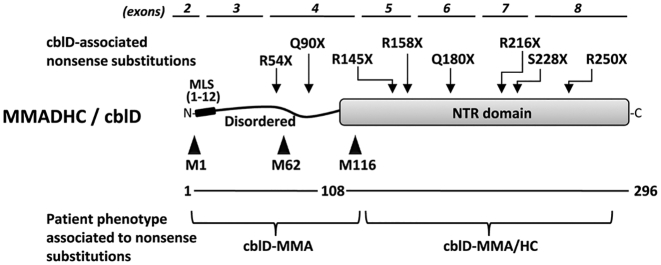

## 1 Introduction

Cobalamin (cbl) or vitamin B12 is one of the most essential enzymatic cofactors in cells, and it is required for normal cellular metabolism. Only a few bacteria and archaea can produce cbl, and mammals lack the enzyme required for *de novo* cbl synthesis, making necessary its obtaining from animal products or supplements. Cbl is crucial for human development and survival, and its deficiency can cause a variety of health problems like megaloblastic anemias, respiratory or gastrointestinal alterations, and neurologic dysfunctions with signs of demyelination, developmental delay, movement disorders and hypotonia, among others [[1], [2], [3], [4]]. Inside the cell, cbl is converted into two cofactors, adenosylcobalamin (AdoCbl) and methylcobalamin (MeCbl). AdoCbl enables the conversion in the mitochondria of *L*-methylmalonyl-coenzyme A into succinyl-coenzyme A by the methylmalonyl-coenzyme A mutase (MMUT; EC 5.4.99.2). Succinyl-coenzyme A is an essential metabolite for odd-chain fatty acid and some branched-chain amino acids catabolism, before entering into the Krebs cycle. MeCbl acts as a cofactor for the methionine synthase (MS; 5-methyltetrahydrofolate:L-homocysteine *S*-methyltransferase; EC 2.1.1.13), which catalyzes in the cytosol the synthesis of methionine from homocysteine. MS is reactivated by the methionine synthase reductase ([methionine synthase]-methylcob(*III*)alamin,S-adenosyl-L-homocysteine:NADP+ oxidoreductase; EC 1.16.1.8). Disruptions in these pathways cause several cbl-related metabolic multisystem disorders (cblA to cblG, cblJ, mut), which manifest with elevated levels of methylmalonic acid and/or homocysteine [5]. Among these disorders the cblD-type disorder is a unique very rare disease associated with three distinct biochemical phenotypes: methylmalonic aciduria (MMA), homocystinuria (HC) or combined MMA/HC [6].

**Fig. 1:**
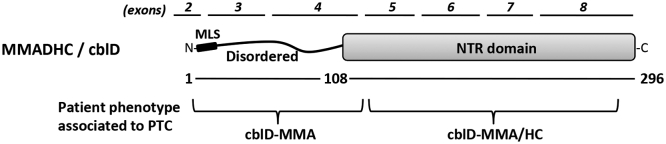
Schematic depiction of MMADHC protein and its targeting by premature termination codons (PTC) in cblD disorder. The N-terminal disordered region (residues 1–108) and the C-terminal Nitro Reductase-like (NTR) domain (residues 109–296) are indicated. MLS, potential mitochondrial leader sequence, (residues 1–12). Amino acid numbering is according to accession NP_056517. The exons encoding the different MMADHC regions are shown at the top. The regions targeted by PTC from patients with MMA or MMA/HC phenotypes are shown at the bottom.

MMADHC (methylmalonic aciduria and homocystinuria type D protein; cblD) protein is essential for the correct trafficking of cbl inside the cell, and inborn mutations at the *MMADHC* gene cause the distinct phenotypic manifestations of cblD disorder with an autosomal recessive pattern of inheritance [7,8]. Evidence indicates that MMADHC constitutes the node of the cytosolic and mitochondrial cbl metabolic pathways [6,9], acting as an adaptor protein for MMACHC (cblC) in multiprotein complexes with MS and MS reductase [[10], [11], [12], [13], [14]]. MMADHC is a 296-amino acids (32.9 kDa) protein with an N-terminal disordered region (amino acids 1–107) containing a potential mitochondrial leader sequence (MLS; amino acids 1–12), and a C-terminal Nitro Reductase-like domain (NTR; amino acids 108–296) [7,12,15]. MMADHC provides a sulfur ligand to cbl bound to MMACHC [16], and the MMADHC NTR domain enhances the oxidation of cbl [15]. Genetic alterations in the *MMADHC* gene cause cblD-MMA, cblD-HC or cblD-MMA/HC, depending on the nature and localization of the mutation. Mutational/functional analysis has delineated that the MMADHC N-terminal disordered region is essential for AdoCbl synthesis, whereas the C-terminal NTR domain is essential for MeCbl synthesis. In this regard, cblD-MMA/HC phenotype has been associated with MMADHC C-terminal truncations caused by premature termination codons (PTC), frameshift, or splicing-defect mutational events, whereas cblD-MMA phenotype has been associated with N-terminal truncations and missense mutations at the C-terminal NTR domain [7,17,18] (Fig. 1).

Several genetic alterations identified in *MMADHC* gene are nonsense single-nucleotide substitutions resulting in PTC. This opens the possibility of using induced translational PTC readthrough as a therapeutic approach to reconstitute the expression and function of full-length MMADHC proteins in specific cblD patients. Translational PTC readthrough can be induced by aminoglycoside and non-aminoglycoside compounds, which interfere with biosynthetic protein translation and increase the error in the decoding process at sites of premature termination of protein biosynthesis. As a consequence, amino acids encoded by near-cognate codons are incorporated at PTC positions, and a full-length protein is synthesized with variable efficiency, depending on the specific PTC and the PTC readthrough inducer [[19], [20], [21], [22]].

The purpose of this study was to characterize the biochemical features and subcellular localization of MMADHC variants derived from PTC mutations, and to analyze their translational readthrough in response to PTC inducers. Our results illustrate how specific disease-associated PTC affect MMADHC protein translation initiation, and show that induced PTC readthrough is effective to reconstitute full-length expression of specific MMADHC PTC variants.

## 2 Materials and methods

### 2.1 Plasmids and mutagenesis

pRK5 HA-MMADHC has been previously described [23]. pRK5 MMADHC-GFP was generated by direct subcloning of MMADHC-GFP cDNA, from pCMV3 MMADHC-GFP Spark (Sino Biologicals), into the pRK5 plasmid [24]. Human MMADHC nucleotide and protein entries are NM_015702 and NP_056517, respectively). Nucleotide substitutions were performed by PCR oligonucleotide-directed site directed mutagenesis, as described [25]. All constructs and mutations were confirmed by digestion with specific restriction enzymes and DNA sequencing.

### 2.2 Cell lines, cell cultures, and transfections

Simian kidney COS-7 cells (ATCC) were grown at 37 °C and 5% CO_2_ in DMEM (*Dulbeccos's Modified Eagle's Medium*, Lonza) with 5% heat-inactivated fetal bovine serum (FBS), 1 mM l-glutamine, 100 U/ml penicillin and 0.1 mg/ml streptomycin. Semi-confluent cells were incubated 16–24 h with GeneJet™ transfection reagent (SignaGen Laboratories) and the appropriate cDNA plasmids, according to the manufacturer specifications. Medium was changed 24 h after transfection and cells were processed 48 h after.

### 2.3 Readthrough induction

The PTC readthrough inducers used were: G418 (Geneticin; Merck Millipore, #345810; 200 μg/ml, or the indicated dose in Fig. 4), gentamicin (Merck Millipore, #345815; 800 μg/ml), and amikacin (Merck Sigma Aldrich, #A03680; 2 mg/ml). The inducers were added 24 h after changing transfection media, and cells were incubated for additional 24 h. For semi-quantitative determination of PTC readthrough efficiency, the full-length MMADHC-GFP protein bands were quantified using an Image studio™ software with Odyssey® CLx Imaging System (LI-COR Biosciences). PTC readthrough efficiency was determined as the percentage of each full-length MMADHC-GFP PTC variant translated under PTC readthrough-inducing conditions compared to the translation of MMADHC-GFP wild type (100%). All quantifications are shown as the mean ± s.d. from at least two independent experiments.

### 2.4 Fluorescence microscopy, antibodies, and immunoblot

For protein localization experiments by fluorescence microscopy, COS-7 cells expressing MMADHC-GFP were directly visualized using a Leica DMI 60000B fluorescent microscope. Cell nuclei were stained with Hoechst 33342 (ThermoFisher Scientific). For immunoblot, COS-7 cells were processed as described [26]. Briefly, cells were washed with PBS and lysed in ice-cold M-PER lysis buffer (ThermoFisher Scientific) supplemented with PhosSTOP phosphatase inhibitor and complete protease inhibitor cocktails (Roche). The lysate was clarified by centrifugation at 15,200*g* for 10 min at 4 °C. Supernatant (whole-cell extract) was collected and 50–100 μg of protein was mixed with NuPAGE LDS sample buffer (4×) (ThermoFisher Scientific) and boiled. Samples were resolved by 10%- or 12%-SDS-PAGE under reducing conditions, and transferred to PVDF membranes (Immobilon-FL, Millipore). Antibodies used were: rabbit polyclonal anti-GFP (G1544, Sigma Aldrich), mouse monoclonal anti-HA (12CA5, ThermoFisher Scientific), and mouse monoclonal anti-GAPDH (6C5, Santa Cruz Biotechnology). Secondary antibodies conjugated with fluorochrome were anti-rabbit or anti-mouse IgG-IRDyeR 800CW (or IgG-Alexa FluoR 680) (LI-COR Bioscience).

## 3 Results

### 3.1 MMADHC N-terminal truncated proteoforms

**Fig. 2:**
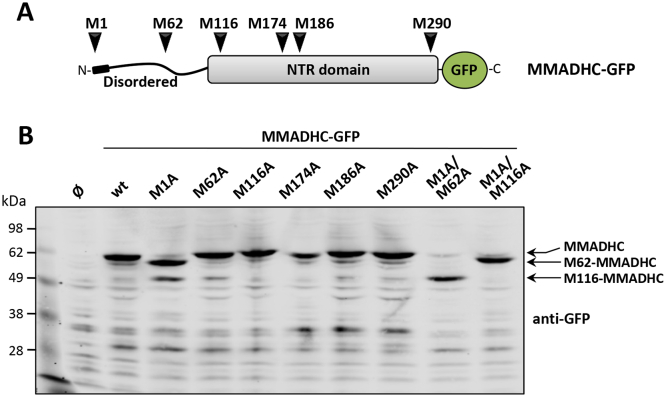
Usage of initiation Met codons in MMADHC protein translation. A. Schematic depiction of the location of the different Met from MMADHC amino acid sequence. B. COS-7 cells were transfected with plasmids encoding MMADHC-GFP wild type (wt) or Met to Ala mutations, as indicated, and cell lysates were resolved in 10% SDS-PAGE gel and analyzed by immunoblot with anti-GFP antibody. Ø, empty vector. The migrations of MMADHC-GFP, M62-MMADHC-GFP, and M116-MMADHC-GFP are indicated, and correspond to the expected molecular weights.

Alternative usage of initiation Met codons during MMADHC protein translation has been found in cells from cblD patients carrying specific PTC mutations at *MMADHC* gene [18]. To understand how genetic variability could affect MMADHC protein translation and stability, we performed ectopic expression experiments using COS-7 cells transfected with recombinant human MMADHC forms bearing epitope tags at its N- or C-terminus. First, the expression of a wild type MMADHC form fused C-terminally to green fluorescent protein (MMADHC-GFP) was monitored by immunoblot using anti-GFP antibody, in comparison with MMADHC-GFP mutated versions in which each MMADHC Met (M1, M62, M116, M174, M186, and M290; Fig. 2A) was substituted individually by Ala (M1A, M62A, M116A, M174A, M186A, and M290A mutations) to abrogate potential translation initiation sites. As shown, upon mutation of the Met1 (M1A mutation), two MMADHC-GFP proteoforms were detected corresponding to the usage of Met62 and Met116 as translation initiation Met codons, as demonstrated using the double mutations M1A/M62A and M1A/M116A. We have named this N-terminal truncated proteoforms with a prefix indicating the initiation Met (M62-MMADHC, and M116-MMADHC) (Fig. 2B).

### 3.2 Protein stability of PTC C-terminal truncated MMADHC variants

**Fig. 3:**
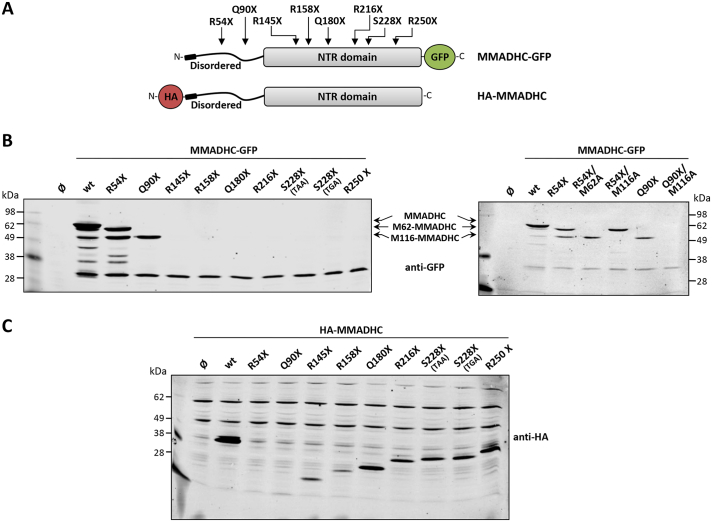
Protein translation of MMADHC PTC mutations associated to cblD disorder. A. Schematic depiction of the location of the different cblD-associated MMADHC mutations causing PTC. B. COS-7 cells were transfected with plasmids encoding MMADHC-GFP wild type (wt) or PTC mutations, as indicated, and cell lysates were resolved in 10% SDS-PAGE gel and analyzed by immunoblot with anti-GFP antibody. C. COS-7 cells were transfected with plasmids encoding HA-MMADHC wild type (wt) or PTC mutations, as indicated, and cell lysates were resolved in 12% SDS-PAGE gel and analyzed by immunoblot with anti-HA antibody. Ø, empty vector. In B, the migrations of MMADHC-GFP, M62-MMADHC-GFP, and M116-MMADHC-GFP are indicated, and correspond to the expected molecular weights.

Next, we asked whether PTC described in cblD patients could give rise to specific truncated MMADHC variants. Five different *MMADHC* gene codons targeted by nonsense mutations creating PTC (R54X [TGA], R158X [TGA], Q180X [TAA], S228X [TGA], and R250X [TGA] mutations) have been described in the literature in cblD patients [7,[27], [28], [29]]. In addition, four other *MMADHC* PTC mutations have been reported in databases (Q90X [TAG], R145X [TGA], R216X [TGA], and S228X [TAA] mutations) (Table 1; ref. [30–32]). The disease-associated PTC MMADHC mutations are mainly found at the MMADHC C-terminal domain, with two PTC mutations (R54X and Q90X) found at the disordered MMADHC N-terminal region (Fig. 3A). To analyze the expression of PTC MMADHC proteoforms, PTC mutations were generated in a MMADHC protein background containing N-terminal hemagglutinin (HA) or C-terminal GFP tags (Fig. 3A), and immunoblot analysis was performed using anti-GFP (Fig. 3B) or anti-HA (Fig. 3C) antibodies. R54X mutation resulted in the translation of two major N-terminal truncated MMADHC-GFP proteoforms, whereas Q90X mutation rendered one N-terminal truncated MMADHC-GFP proteoform. Further mutational analysis using the double mutations R54X/M62A, R54X/M116A, and Q90X/M116A, demonstrated that Met62 and Met116 are the initiation Met codons of these proteoforms. No proteoforms starting at the other MMADHC Met were consistently detected (Fig. 3B). This demonstrates that reinitiation of translation takes place in MMADHC at Met62 and Met116 upon termination of translation by a PTC at Arg54, and at Met116 upon termination of translation by a PTC at Gln90. These results also show that the Met located in the MMADHC amino acid sequence after Met116 are not productive to generate MMADHC truncated proteoforms, even in the presence of upstream PTC at the MMADHC sequence.

**Table 1:** Germline *MMADHC* PTC mutations^1^.

Mutation^2^	Amino acid substitution^2^	Codon^3^	Exon	Patient phenotype^4^	Reported in databases^5^				Ref.^6^
					HGMD	ClinVar	ExAC/gnomAD	Exome Variant	
c.160C > T	p.(Arg54Term)^7^ / R54X	TCT TGA ACA	4	cblD MMA	+	+			[7]
c.268C > T	p.(Gln90Term) / Q90X	TCA TAG AAG	4				+		
c.433A > T	p.(Arg145Term) / R145X	GCC TGA GTA	5				+		
c.472C > T	p.(Arg158Term) / R158X	CTG TGA AAA	5	cblD MMA/HC	+	+	+		[27]
c.538C > T	p.(Gln180Term) / Q180X	ACA TAA AAA	6	cblD MMA/HC	+			+	[29]
c.646C > T	p.(Arg216Term) / R216X	CTT TGA GCT	7				+		
c.683C > A c.683C > G	p.(Ser228Term) / S228X	CCA TAA TCT CCA TGA TCT	7	cblD MMA/HC	+		+ +		[28]
c.748C > T	p.(Arg250Ter)^8^ / R250X	TAC TGA CAT	8	cblD MMA/HC	+	+	+		[7,23]

1 Nonsense mutations generating premature termination codons (PTC) at *MMADHC* gene and reported in the literature or in databases are indicated. Frameshift mutations that create downstream PTC are not included.

2 Nucleotide and amino acid substitutions are indicated following HGVS recommended nomenclature, as well as with single-letter code amino acid nomenclature. Nucleotide and amino acid numbering corresponds to accessions NM_015702.3 and NP_056517.1, respectively.

3 The PTC is underlined and flanked by the adjacent codons.

4 MMA, Methylmalonic aciduria; HC, Homocystinuria. Note that in some cases the patient phenotype is not available

5 HGMD, Human Gene Mutation Database [32]; ClinVar (NCBI) [30]; Exome Aggregation Consortium (ExAC), Genome Aggregation Database (gnomAD) (Broad Institute) [31]; Exome Variant Server (NHLBI Exome Sequencing Project).

6 Original references (to the best of our knowledge) for mutation-encoding variants from patients are provided.

7 Heterozygous carrier: c.160C>T / c.307_324dup.

8 Heterozygous carrier in ref [23]: c.748C>T / c.438_442del

Next, we analyzed the expression of the N-terminal portion of the MMADHC PTC variants using an anti-HA antibody, which recognizes the MMADHC N-terminal tag. As shown, MMADHC C-terminal truncations of proportionally increased size could be detected starting at R145X up to the R250X PTC MMADHC variants (Fig. 3C).

In summary, our experiments indicate that MMADHC Met62 and Met116 are used to reinitiate protein translation upon R54X and Q90X PTC mutations, generating two distinct N-terminal MMADHC truncated proteins (M62-MMADHC and M116-MMADHC). In addition, we detected the expression of MMADHC C-terminal truncations caused by PTC located at the MMADHC NTR domain.

### 3.3 Induction of translational PTC readthrough on MMADHC PTC variants

**Fig. 4:**
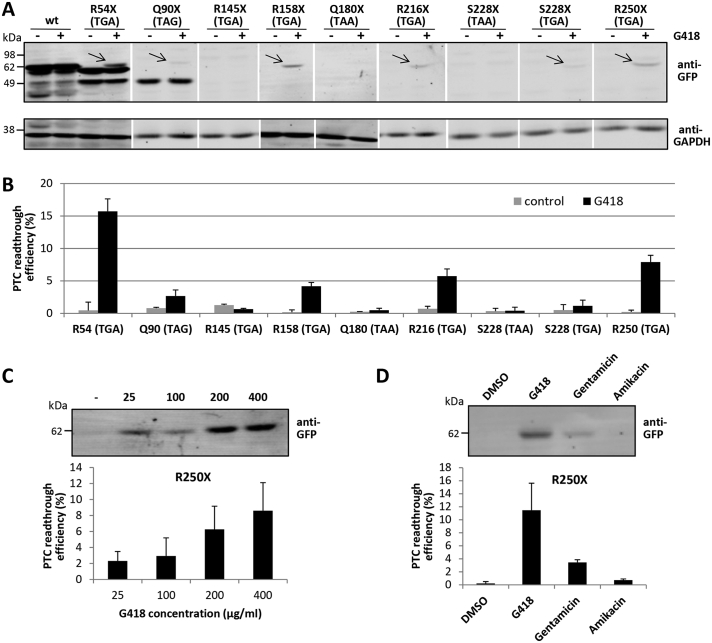
Readthrough response of MMADHC PTC mutations associated to cblD disorder. COS-7 cells were transfected and analyzed as in Fig. 3. A. Cells expressing MMADHC-GFP wild type (wt) or the different cblD-associated PTC mutations were kept untreated or were treated for 24 h with G418 (200 μg/ml), and cell lysates were analyzed by immunoblot with anti-GFP antibody. Arrows indicate the migration of the full-length MMADHC-GFP proteins. Anti-GAPDH is shown as a loading control. B. Semi-quantification of PTC readthrough efficiency of MMADHC-GFP PTC variants upon G418 treatment, from cells treated as in A. C. Cells expressing MMADHC-GFP R250X were treated for 24 h with the indicated concentrations of G418, and cell lysates were analyzed by immunoblot with anti-GFP antibody. A representative experiment is shown at the top, and semi-quantification is shown at the bottom. D. Cells expressing MMADHC-GFP R250X were treated for 24 h with different PTC readthrough inducers (G418, 200 μg/ml; Gentamicin, 800 μg/ml; Amikacin, 2 mg/ml), and cell lysates were analyzed by immunoblot with anti-GFP antibody. A representative experiment is shown at the top, and semi-quantification is shown at the bottom. In B, C, and D, data are shown as the mean ± s.d. of PTC readthrough efficiency from at least two independent experiments, calculated as described under Materials and Methods.

PTC recognition during protein translation can be interfered using specific PTC readthrough-inducing compounds, which permits the synthesis of a full-length protein. We tested the efficiency of PTC readthrough inducers to reconstitute the expression of full-length PTC MMADHC-GFP mutations ectopically expressed in COS-7 cells. Fig. 4A shows representative experiments of PTC readthrough of the different MMADHC PTC variants (Table 1), using as PTC readthrough inducer the aminoglycoside G418 (geneticin). Fig. 4B shows the semi-quantification of the readthrough efficiency for each PTC variant. As shown, MMADHC full-length expression was achieved from MMADHC PTC variants upon readthrough induction in a PTC-dependent manner, ranging from 0 to 20% expression with respect to MMADHC wild type. TGA PTC codons responded more efficiently than TAG or TAA, in agreement with previous PTC readthrough studies [33], with R54X and R250X displaying the higher PTC readthrough responses (16% and 8% expression, respectively). In the case of R54X, the amount of M62-MMADHC and M116-MMADHC N-terminal truncation proteoforms generated upon PTC readthrough induction were decreased concomitantly to the generation of full-length MMADHC. Dose-response of G418 PTC readthrough of MMADHC R250X variant is shown in Fig. 4C, and the PTC readthrough response of MMADHC R250X to other aminoglycosides, including gentamicin and amikacin [34], is illustrated in Fig. 4D. As shown, G418 and gentamicin reconstituted full-length MMADHC R250X expression at variable extent (11.5% and 3.5% expression, respectively, with respect to MMADHC wild type), whereas amikacin did not induce detectable PTC readthrough. In conclusion, PTC-readthrough is successfully achieved in specific PTC MMADHC mutations, resulting in the reconstitution at a variable extent of the expression of full-length MMADHC proteins.

**Fig. 5:**
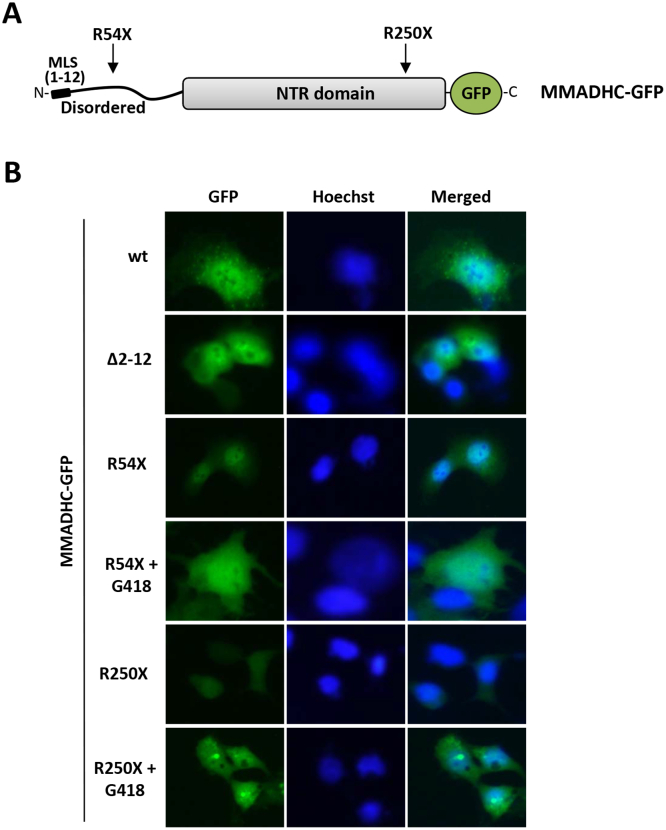
Subcellular localization of MMADHC variants upon PTC-readthrough induction. A. Schematic depiction of the different MMADHC-GFP proteins analyzed by immunofluorescence. B. COS-7 cells were transfected with plasmids encoding MMADHC-GFP wild type (wt) or PTC mutations (R54X, R250X), in the presence or in the absence of G418, as indicated. A MLS-deleted variant (Δ2-12) was also included. Nuclei were stained with Hoechst (blue), and cells were directly visualized by standard fluorescence microscopy. MMADHC-GFP (green), but not MMADHC-GFP Δ2-12 or MMADHC-GFP R54X, displayed a punctate staining. Note in the left panel the punctate expression pattern in MMADHC-GFP R250X upon G418 PTC-readthrough induction.

We further studied by fluorescence microscopy the expression and subcellular localization of the R54X and R250X MMADHC mutations under control conditions and upon PTC readthrough induction, using MMADHC-GFP ectopically expressed in COS-7 cells. In these experiments, a MMADHC variant lacking the N-terminal MLS (MMADHC Δ2-12) was also used. Wild-type MMADHC protein showed a punctate nucleo-cytosolic localization, whereas MMADHC Δ2-12 displayed a more diffuse distribution and lacked the punctate staining (Fig. 5). MMADHC R54X displayed a predominant non-punctate distribution both under control and readthrough induction conditions, reflecting the abundant expression of M62-MMADHC and M116-MMADHC, which lack the MLS. MMADHC R250X expression was not detected under control conditions, but noticeably staining was observed upon G418 incubation, displaying a punctate staining pattern in many of the transfected cells (Fig. 5). This indicates readthrough -induced reconstitution of expression and subcellular localization of full-length MMADHC-GFP. Together, these findings illustrate the differential subcellular localization of wild type MMADHC and MMADHC protein variants lacking the N-terminal region, and confirm the reconstitution by induced PTC readthrough of full-length expression of MMADHC PTC variants in intact cells.

## 4 Discussion

Mutations generating PTC in disease-associated genes represent the single-nucleotide substitutions with a higher pathogenic potential, since they produce, in most of the cases, unstable and/or unfunctional truncated proteins. The metabolic cblD-type disorder is a very rare disease in which the *MMADHC* gene is targeted by PTC mutations with relative high frequency. Here, we have characterized the MMADHC proteoforms generated by disease-associated *MMADHC* PTC, and disclosed the potential of translational PTC readthrough strategies to reconstitute full-length MMADHC protein expression from these PTC-targeted gene variants. It should be noted that recombinant tagged forms of MMADHC protein have been used in our experiments to facilitate MMADHC detection.

Our MMADHC biochemical expression analysis confirms the alternative initiation of protein translation during biosynthesis of MMADHC harboring N-terminal PTC mutations [18], and unequivocally identifies Met62 and Met116 as the alternatively used initiation Met codons during translation of MMADHC R54X and Q90X PTC mutations, rendering the proteoforms M62-MMADHC and M116-MMADHC. Furthermore, our analysis shows that mutation of Met1 (M1A mutation) also results in the synthesis of M62-MMADHC and Met116-MMADHC. This is relevant since a *MMADHC* M1R homozygous mutation (c.2 T > G) has been found in cblD patients with MMA isolated phenotype [35]. Additional *MMADHC* frameshift mutations have been reported generating PTC at the N-terminal MMADHC region [7,18,36], which will also likely use alternative initiation of translation at Met62 and/or Met116. It can be hypothesized that patients harboring these N-terminal MMADHC mutations express M62-MMADHC and M116-MMADHC. Nonsense-mediated alternative translation initiation has been described before, in some cases in association with disease-associated mutations that create a PTC, suggesting that reinitiation of translation after a PTC is a general mechanism [[37], [38], [39]]. It has been proposed that the PTC must be relatively close to both the canonical upstream initiation site and to the downstream reinitiation site, although there are variations in the reported minimal distances between these elements [38,40,41]. In our analysis, reinitiation at MMADHC Met174 (or the rest of downstream Met) from MMADHC R54X or Q90X was not detected. In addition, no reinitiation was detected at Met174 (or the rest of downstream Met) from PTC located immediately upstream, including those at positions Arg145 (R145X mutation) and Arg158 (R158X mutation). It is conceivable that M62-MMADHC and M116-MMADHC, which possess a complete NTR domain, are stable proteins with potential functionality [15]. On the other hand, initiation at Met downstream Met116 would generate N-terminal truncated NTR domains, which are likely to lack function. This is in agreement with the phenotypes shown by patients harboring MMADHC N-terminal truncated mutations [7,17,18]. In addition, we have found variable expression of MMADHC C-terminal truncated forms in the variants targeted by PTC downstream of Gln90, suggesting the existence of MMADHC proteoforms with NTR C-terminal truncated domains. Since most of these MMADHC C-terminal truncated forms lack a large portion of the NTR domain, they are likely to have impaired binding to MMACHC and function. Interestingly, the MMADHC R250X mutation, a prevalent PTC mutation associated to late-onset cblD MMA/HC disorder [23], generates an almost complete NTR domain, but it lacks the Cys261 which acts as the sulfur ligand to cbl bound to MMACHC [16]. This may help to understand the specific late-onset phenotype associated to the R250X mutation, and opens the possibility that this mutation is not completely deleterious for all MMADHC functions during early development. Further work is required to ascertain the molecular mechanisms that drive MMADHC protein variants stability and function in cells.

Our PTC readthrough analysis on the collection of *MMADHC* PTC mutations associated to cblD disorder has unveiled the potential to restore expression and subcellular localization of full-length MMADHC in specific groups of patients. Experiments in a genuine genomic background are required to reinforce these findings in a more physiological context. Several *MMADHC* PTC mutations, including R54X, R158X, R216X, and R250X TGA mutations, displayed a detectable PTC readthrough response to the aminoglycoside G418. On the other hand, the *MMADHC* PTC TAA mutations, including Q180X and S228X, did not respond to G418 PTC readthrough induction. This is in agreement with the reported differential PTC readthrough efficiency depending of the PTC type (TGA > TAG>TAA), which is also affected by the local nucleotide sequences close to the PTC [33,42,43]. Importantly, in the case of MMADHC R54X, the PTC readthrough-induced expression of full-length MMADHC was accompanied by a decrease in the synthesis of the pathogenic M62-MMADHC and M116-MMADHC proteoforms. In the case of MMADHC R250X, the MMADHC PTC variant more frequently found in patients [23], partial restoration of expression and subcellular location was achieved upon readthrough induction.

Although newborn screening, where available, is helping in the early diagnosis of cbl-related disorders and its appropriate pharmacological therapeutic treatment, the clinical benefits of early treatment in cblD defect remain uncertain [44,45]. There is limited experience with OH-Cbl treatment in cblD-MMA/HC disease, but there seems to be a response to treatment in some patients [[44], [45], [46]], and complementary treatments could help to improve patient welfare. PTC readthrough reconstitution has been explored for stop codon-mutated *MMUT* [47,48], and the non-aminoglycoside PTC readthrough inducer Ataluren (PTC124) has been tested in a clinical trial (NCT01141075) on MMA patients with PTC mutations in the *MMUT* gene or in the genes *MMAA* or *MMAB*, involved in conversion of cbl to AdoCbl. Our study addresses the potential of readthrough to counterbalance MMADHC deficiency in cblD patients, and emphasizes the importance of individual genetic and biochemical analysis of MMADHC from each patient, not only to obtain an accurate genetic diagnosis that guide standard interventions, but also to provide information on the actionability by alternative precision therapies. This is well illustrated by our findings on the PTC-specific response to MMADHC expression restoration achieved upon PTC readthrough induction by the aminoglycoside G418. Since many PTC readthrough inducers experimentally used (including G418) display undesired toxicity, searching for highly effective and non-toxic PTC-readthrough-inducing compounds is currently under intense scrutiny in a variety of human diseases, including inborn metabolic diseases [49,50]. In addition, the identity of the amino acid incorporated at the PTC position during readthrough is variable [51], requiring the analysis of protein stability and function of MMADHC restored upon PTC readthrough to further inform on the potential therapeutic efficacy of this approach for cblD disorder.

The degradation of mRNAs harboring PTC is triggered by nonsense-mediated mRNA decay (NMD) mechanisms [52,53]. Importantly, PTC readthrough not only reconstitutes the expression of full-length proteins whose encoding genes are targeted by PTC, but also inhibits NMD of their transcripts [54]. In the case of specific MMADHC PTC mutations, NMD has been shown to be partially reverted by the NMD inhibitor emetine [18]. We hypothesize that low toxicity PTC readthrough inducing agents, alone or in combination with NMD inhibitors, could be beneficial for a group of cblD patients harboring specific PTC mutations.

## Declaration of Competing Interest

The authors have no conflict of interest to declare.
